# Effect of Social Media on Child Obesity: Application of Structural Equation Modeling with the Taguchi Method

**DOI:** 10.3390/ijerph15071343

**Published:** 2018-06-26

**Authors:** Datis Khajeheian, Amir Mohammad Colabi, Nordiana Binti Ahmad Kharman Shah, Che Wan Jasimah Bt Wan Mohamed Radzi, Hashem Salarzadeh Jenatabadi

**Affiliations:** 1 Department of Media Management, Faculty of Management, University of Tehran, Tehran 141556311, Iran; 2 Department of Business Management, Faculty of Management and Economics, Tarbiat Modares University, Tehran 1439813141, Iran; colabi@modares.ac.ir; 3 Department of Library and Information Science, Faculty of Computer Science & Information Technology, University of Malaya, Kuala Lumpur 50603, Malaysia; dina@um.edu.my; 4 Department of Science and Technology Studies, Faculty of Science, University of Malaya, Kuala Lumpur 50603, Malaysia; jasimah@um.edu.my (C.W.J.B.W.M.R.); jenatabadi@um.edu.my (H.S.J.)

**Keywords:** complex analysis, childhood obesity modeling, public health study, overweight

## Abstract

Through public health studies, specifically on child obesity modeling, research scholars have been attempting to identify the factors affecting obesity using suitable statistical techniques. In recent years, regression, structural equation modeling (SEM) and partial least squares (PLS) regression have been the most widely employed statistical modeling techniques in public health studies. The main objective of this study to apply the Taguchi method to introduce a new pattern rather than a model for analyzing the body mass index (BMI) of children as a representative of childhood obesity levels mainly related to social media use. The data analysis includes two main parts. The first part entails selecting significant indicators for the proposed framework by applying SEM for primary and high school students separately. The second part introduces the Taguchi method as a realistic and reliable approach to exploring which combination of significant variables leads to high obesity levels in children. AMOS software (IBM, Armonk, NY, USA) was applied in the first part of data analysis and MINITAB software (Minitab Inc., State College, PA, USA) was utilized for the Taguchi experimental analysis (second data analysis part). This study will help research scholars view the data and a pattern rather than a model, as a combination of different factor levels for target factor optimization.

## 1. Introduction

In 2016, the World Health Organization (WHO) reported that around 170 million children below the age of 18 were suffering from obesity and overweight [1]. Some researchers consider this concern regarding children as one of the greatest and most crucial threats to public health in the last twenty years [2,3]. Obesity is now acknowledged as a severe hazard to society due to its rapidly expanding prevalence [4]. Consequently, focus should be geared toward preventing child overweight and obesity.

Previous studies on childhood overweight and obesity modeling illustrate that the main factors are family environment and children’s lifestyle. A number of studies indicate that family environment is an important determinant of children’s lifestyle [5] and both have an impact on child BMI level. Hence, information on children’s lifestyle is often collected based on household environment surveys. Decision makers are able to use such information to allocate resources prudently when planning activities aimed at improving the overall lifestyle of children and adolescents. Previous studies specify that several factors are associated with the children’s lifestyle index, including parental lifestyle [6] and parental socioeconomic status [7,8,9]. In general, family socio-economic status [10,11,12], child-feeding behavior [13], children’s physical activity [14,15,16] and food intake [17] are the most familiar factors involved in childhood obesity modeling. Moreover, some researchers added television viewing and computer use [5,18] in studies to develop models to analyze children’s health and lifestyle. With the current development of digital technologies, especially mobiles and tablets, the daily average use has been increasing among children, youth and adults. Progressively prevalent social media (e.g., Facebook, Instagram, Twitter, YouTube, WeChat, etc.) have altered many aspects of our daily lives. Consequently, the structure of children’s lifestyle in terms of social media usage has also changed.

While the main feature of Web 1.0 was cognition and of Web 2.0 connection, the main feature of social media today is the provision of a space for cooperation [19]. Cooperation promotes the concept of online communities based on social participation [20]. Waring et al. [21] showed that in terms of weight loss, online communities provide social support for users to engage with others in weight loss activities. A survey by Pagoto et al. [22] showed that for 100 adults, Twitter followers are more significant sources of encouragement for weight loss rather than family and friends. Higher positive feedback was received from Twitter followers, and thus greater attempts at weight loss have been reported. Evans et al. [23] found that bloggers who report their weight loss goals on their weblogs feel accountable to their readers and thus increase related activities. Waring et al. [21] argued that “even in the absence of interaction, adults may find following others attempting to lose weight, obesity experts, and related organizations’ social media feeds for information helpful to their weight loss efforts.” Gruver et al. [24] suggested that social media peer groups may offer a promising new way of providing families with the knowledge, strategies and support they need to adopt obesity prevention behaviors.

In contrast, social media can also have negative effects. Mazur et al. [25] showed that children who use social media more are at greater risk of sleep disturbance that leads to obesity. They reported a 40% decrease in obesity in children whose parents limit their screen-time than in children without such limitation. Content that users consume on social media is another important subject. Holmberg [26] discussed the persuasive effect of food marketing through social media on obesity. He argued that social media can be used proactively in clinical practice, to inspire people to behave in healthier ways, such as cook at home and consume healthier diets. For example, users can share photos of their kitchen and healthy meals while clinical staff provide feedback. Nonetheless, Swindle et al. [27] believe that digital technology delivers solutions and can facilitate nutrition educators to connect with at-risk populations. The Internet, for instance, has long been the most extensive supply of health data [28]. Studies report the successful use of social media like Facebook for educating parents on child obesity prevention [29]. However, to develop a research framework for obesity Hall et al. [30] suggested factors within broader social and environmental contexts that contribute to the risk of obesity. A current study by Huang et al. [4] proved that daily average technology use has a significant effect on children’s weight. Moreover, they found that the relationship between family socio-economic status, technology use by children and children’s weight is stronger in the obesity model than the non-obesity model. However, there are insufficient studies on the impact of daily social media use on children’s BMI level according to different educational levels from primary to high school.

Iran has been facing the problem of child obesity and overweight in the last two decades. A study by Soltani et al. [31] demonstrated that more than 18% of children in elementary school (6–11 years old) exhibit the highest prevalence and risk of obesity and overweight. The challenges of obesity and overweight during childhood and the teenage years have negative and dangerous ramifications for premature mortality and morbidity as well as physical disability later in adulthood [32]. The association between obesity/overweight and eating habits while watching television was studied by Ghobadi et al. [33] in Iran. Unfortunately, there are very few studies in Iran that investigate the impact of child behavior like technology use, average sleep time and physical activity considering the family environment and especially the family socio-economic status, child-feeding behavior and children’s BMI level. Nevertheless, research on the simultaneous integration of the interrelationships among three well-known concepts, i.e., family socio-economic status, family child-feeding behavior and children’s healthy and unhealthy food intake into one model remains scarce.

Descriptive statistics [34], Analysis of Variance (ANOVA) [35,36], neural networks [37], regression [38,39,40,41,42] and fuzzy sets [43] are popular statistical methods of analyzing obesity. Among these statistical modeling techniques, regression (bivariate or multivariate) is the most widely used to analyze child obesity modeling. In recent decades, a new modeling application, structural equation modeling (SEM), has been applied in child obesity analysis studies [44]. With the SEM technique it is possible to hypothesize all kinds of interactions and associations among research variables in a single causal framework. This approach is supportive for research scholars to better understand the concept of latent variables and their action within the model and output interpretation. This method has been employed in a wide range of studies, especially in public health [45,46,47]. Ordinarily, SEM has a robust capability of combining both measurement variables (observed) and constructs (non-observed) with the causal relations among them, which leads to a dependent (or output) variable. SEM has served as a better alternative to general linear models (GLMs), such as linear or non-linear regression, factor analysis, etc. 

Statistical modeling like regression, SEM, partial least squares (PLS) or even mathematical modeling techniques including neuro-fuzzy inference systems can determine the significant independent (input) variables. However, they are not able to illustrate which levels or categories of independent variables lead to higher, lower or nominal dependent (output) variable rates. Therefore, statistical or mathematical modeling techniques cannot answer the question: what level or category of independent variables leads to higher or lower dependent variables? The Taguchi method can answer this question. This method has been applied in various engineering studies [48,49] but Taguchi experimental analysis lacks in public health studies. Therefore, the main purpose of this study is to introduce the Taguchi method to identify a pattern that combines input variables that lead to child obesity.

## 2. Taguchi Method Structure

The Taguchi method is based on measuring the standard deviation and calculating the variation in the expected value as follows:

1. High standard deviation denotes that the observed values are spread out from the expected value due to noise factors.
2. Lower standard deviation denotes that the observed values are near the expected value on account of noise factors.

It is not easy to control noise factors, but researchers believe that both observed and noise factor values can be controlled by the Signal-to-Noise (SN) ratio. The SN ratio defines the impact of noise factors on performance characteristics and quantifies the variability [50]. Based on optimization type, there are three SN applications, which are defined as follows:

(a) $\mathrm{SN}=\;-10\log\;(\frac{\sum y^{2}}{n})$ if seeking an optimal situation based on a smaller rate.
(b) $\mathrm{SN}\;=\;-10\log\;(\frac{\sum y^{1/2}}{n})$ if seeking an optimal situation based on a larger rate.
(c) $\mathrm{SN}\;=\;10\log\;(\frac{\bar{y}}{s_{y}^{2}})$ if seeking an optimal situation based on a nominal rate.

where *y* is the observed data, *n* is the number of observations, $\bar{y}\;$ is the average of observed data, and $s_{y}^{2}$ is the variance of *y*.

**Figure 1:**
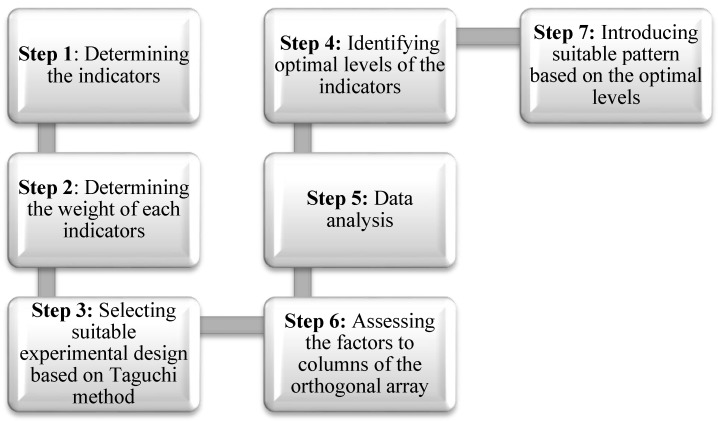
Taguchi method analysis process [51].

Figure 1 shows the analysis process based on the Taguchi method.

## 3. Materials and Methods

### 3.1. Sampling Procedure

The cross-sectional research design was applied in the current study. According to the statistical sampling concept, cross-sectional research design applies any assumed research population sample at one point in time to obtain the essential data. The education system in Iran is divided into two main levels:

- Primary school level: 6 years long, from 6 years old (grade 1) to 11 years old (grade 6),
- High school level: 6 years long, from 12 years old (grade 1) to 17 years old (grade 6).

Both primary school and high school students were considered for the data collection. The data were collected in Tehran, Iran, from 5 September 2017 to 20 February 2018. Tehran has 35 public primary schools and 62 high schools. A combination of cluster and stratified sampling was used in the current study. The data collection contained three steps. The first step entailed cluster sampling, whereby every high school and primary school reflected one cluster. All schools were contacted by phone and email to explain the project objectives and request collaboration for this research. At the end, 12 high schools and 19 primary schools confirmed participation in this project. In the first sampling step where every school was denoted a cluster, a total of 31 clusters (12 + 19 = 31) were obtained. Sixty (60) questionnaires were distributed to every cluster (school). Stratified sampling entailed the second step of the sampling procedure. Here, six strata (every school grade = 1 stratum) were considered in every cluster. Every school has six grades (six strata), and 10 questionnaires were distributed for each stratum. In the third step, random sampling was used to select participants from volunteer parents. Therefore, the sample number was 31 × 6 × 10 = 1860 questionnaires. Eighteen (18) bachelor and master students of management and public health were trained for the data collection phase.

### 3.2. Measuring the Indicators

#### 3.2.1. Obesity Level

In this study, BMI represents the obesity level. Song et al. [52] defined BMI as a measure of relative size based on the mass and height of an individual with the following formula:

(1) $$\;BMI\;\left(Metric\;Method\right)=\;\frac{\left(weight\;in\;kilograms\right)}{height\;in\;meters^{2}}$$

Child BMI is measured in the same way as for adults, except it is then compared to the typical values of other children of the same age. Instead of comparing against overweight thresholds and fixed underweight values, the BMI is compared against a percentile of children of the same gender and age [53]. Table 1 provides the child BMI categories. A BMI below the 5th percentile indicates underweight and above the 95th percentile, obesity. Children with a BMI between the 85th and 95th percentiles are considered overweight [53]. The weight and height information were collected from the students’ health cards. The children’s BMI levels were measured by considering 1, 2, 3, 4, and 5 for ‘underweight,’ ‘normal range,’ ‘at risk,’ ‘moderately obese’ and ‘severely obese.’

#### 3.2.2. Family Socio-Economic Status

In the current study, family socio-economic status was measured in terms of four criteria: father and mother’s age, education, income and work experience, as follows:

- Age: (a) less than 31 years old (value 1); (b) 31–40 years old (value 2); (c) 41–50 years old (value 3); (d) 51–60 years old (value 4); (e) over 60 years old (value 5).
- Education: (a) less than high school (value 1); (b) high school (value 2); (c) diploma (value 3); (d) bachelor (value 4); (e) master or PhD (value 5).
- Job Experience: (a) less than 5 years (value 1); (b) 5 to 10 years (value 2); (c) 11 to 15 years (value 3); (d) 16 to 20 years (value 4); (e) over 20 years (value 5).
- Income: (a) less than 2MT [MT: Million Tomans] per month (value 1); (b) 2 to 3MT per month (value 2); (c) 3 to 4MT per month (value 3); (d) 4 to 5MT per month (value 4); (e) over 5MT per month (value 5).

#### 3.2.3. Food Intake of Children

Kröller and Warschburger [13] introduced seven factors to measure the food intake of children, which is divided into two groups: healthy and unhealthy food intake. This concept was applied in the present work to measure the food intake of children. Healthy food intake includes the consumption of whole grain products (including whole grain rice, pasta, bread, and cereals), fruits (including all kinds of unsweetened fruits, frozen or fresh) and vegetables (including frozen, dry or fresh), whereas unhealthy food intake entails the consumption of fast food (e.g., hot dogs, pizza, and burgers), chips (e.g., nuts, chips, and pretzels), soft drinks (including all types of sweetened beverages) and sweets (e.g., cookies, candies, chocolate, and cake). Every indicator was measured using a five-point scale. The responses obtained were coded as 1 for ‘never’, 2 for ‘rarely, 3 for ‘sometimes’, 4 for ‘mostly’ and 5 for ‘always.’

#### 3.2.4. Family Child-Feeding Behavior

Birch et al. [54] designed the child feeding questionnaire (CFQ) for measuring family child-feeding behavior. The six factors in CFQ are modeling, controlling, pressuring, rewarding, monitoring and restricting. A five-point scale was also applied to measure these indicators. The responses obtained were coded as 1 for ‘never’, 2 for ‘rarely, 3 for ‘sometimes’, 4 for ‘mostly’ and 5 for ‘always.’

#### 3.2.5. Other Factors

In addition to the above factors, the following are also included in the present research framework:

##### Children’s Social Media Use

Social media has been defined by different authors. Kaplan and Haenlein [55] define social media as “a group of internet-based applications that build on the ideological and technological foundations of Web 2.0, and that allow the creation and exchange of UGC”. Khajeheian [56] shows that by advances in technology, social media can be used in various devices, such as mobile-devices, laptops and PCs, tablets, video consoles and even television sets. Doub et al. [57] consider social media as a platform which is accessible through Internet-connected devices including computers, tablets, and smartphones.

In our study, the average number of hours per day that children use social media (e.g., on TV, mobile, tablet, etc.) is divided into four categories: (a) less than 1 h per day (value 1); (b) 1 to 2 h per day (value 2); (c) 2 to 3 h per day (value 3); (d) 3 to 4 h per day (value 4) and (e) more than 4 h per day (value 5).

##### Children’s Physical Activity

The average number of times per week that children do physical activities is coded into four categories: (a) none (value 1); (b) 1 or 2 times per week (value 2); (c) 3 or 4 times per week (value 3); (d) 5 or 6 times per week (value 4) and (e) every day (value 5).

##### Children’s Sleep Amount

The average number of hours per day that children sleep are divided into four categories: (a) less than 6 h per day (value 1); (b) 6 to 7 h per day (value 2); (c) 7 to 8 h per day (value 3); (d) 8 to 9 h per day (value 4) and (e) more than 9 h per day (value 5).

## 4. Results

From 1860 questionnaires distributed (1140 to primary schools and 720 to high schools), 1563 were completed and returned (958 from primary schools and 611 from high schools). 

### 4.1. Descriptive Statistical Analysis

**Figure 2:**
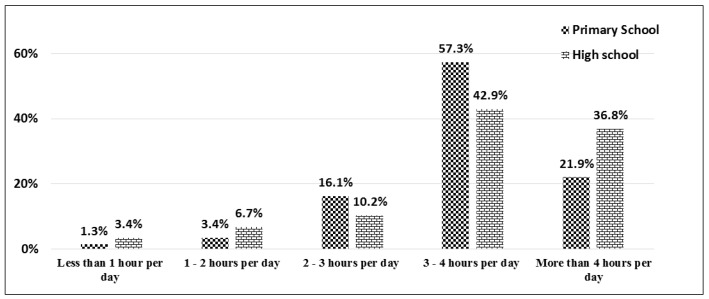
Distribution of *children’s social media use* among primary and high school students.

**Figure 3:**
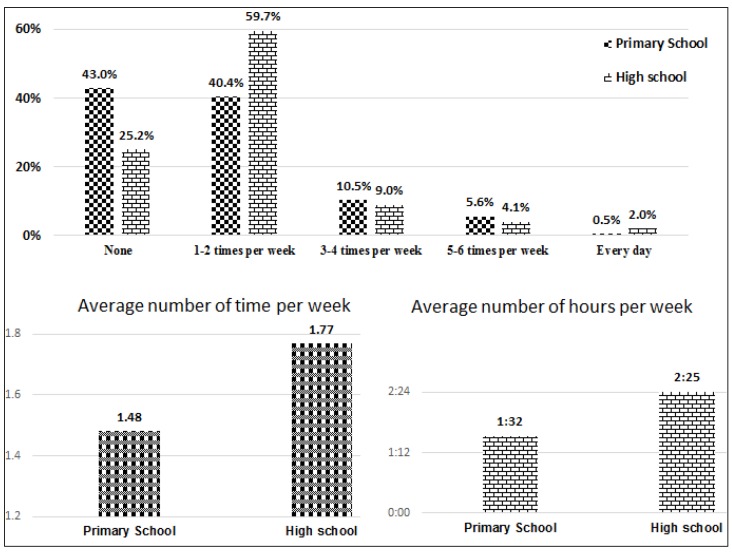
Distribution of *children’s physical activity* among primary and high school students.

**Figure 4:**
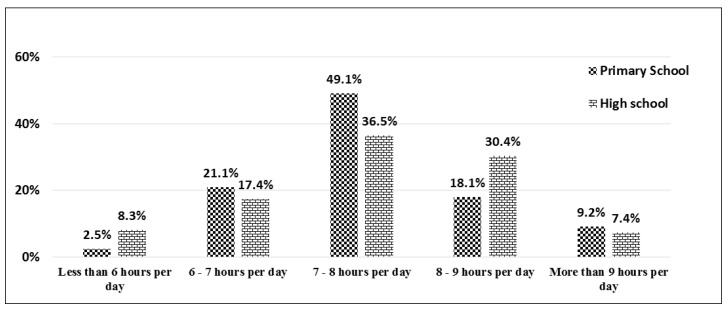
Distribution of *children’s sleep amount* among primary and high school students.

Table 2 and Table 3 and Figure 2, Figure 3 and Figure 4 present the research variable distribution among primary and high school students. Based on Table 2, 12.00% of primary school children (115 out of 958) from the study sample are underweight, 72.55% (695 out of 958) are in the normal range and 15.44% (148 out of 958) are overweight and obese. However, among high school students, 15.06% are underweight, 65.79% are in the normal range, and 19.14% are overweight.

Among the sample population, 57.3% of primary school students and 42.9% of high school students use social media 3–4 h per day. Moreover, 21.9% of primary school students and 36.8% of high school students use social media more than 4 h per day. In general, 79.2% of primary school students and 79.7% of high school students use social media more than 3 h per day (Figure 2).

From 958 primary school students, 43% do no physical activity, 40.4% do physical activity 1–2 times per week, 10.5% do 3–4 times per week, 5.6% do 5–6 times per week, and only less than 1% do physical activity every day. However, among high school students, 25.2% do not exercise, 59.7% do so 1–2 times per week, 9% do 3–4 times per week, 4.1% do 5–6 times per week and around 2% do exercise every day (Figure 3). 

Based on Figure 4, most primary school students (49.1%) and high school students (36.5%) get 7–8 h of sleep per day. More than 18% of primary school students get 8–9 h of sleep per day. However, among high school students, more than 30% get 8–9 h of sleep per day.

Based on Table 3, for the primary school group the fathers’ age categories are 41–50 years old (52.4%), then 31–40 years old (25.9%) and 51–60 years old (12.7%). For the high school group the fathers’ age categories are 41–50 years old (49.8%) and 51–60 years old (36.2%). In the primary school group, 6.2% of mothers are less than 31 years old, 26.6% 31–40 years old, 37.9% 41–50 years old, 25.9% 51–60 years old, and 3.4% over 60 years old. For the high school group, the distribution is: 0.7% (less than 31 years old), 36.8% (31–40 years old), 39.6% (41–50 years old), 21.6% (51–60 years old) and 1.3% (over 60 years old).

The highest education level for parents is diploma. More than 57% of fathers and around 35% of mothers of primary school students have a diploma. These values for high school students are 55% (father) and 32.9% (mother). Almost 27% and 37% of fathers of primary school and high school students have bachelor degrees, and 37% and 33% of mothers have bachelor degrees respectively (Table 3).

### 4.2. SEM Analysis

**Figure 5:**
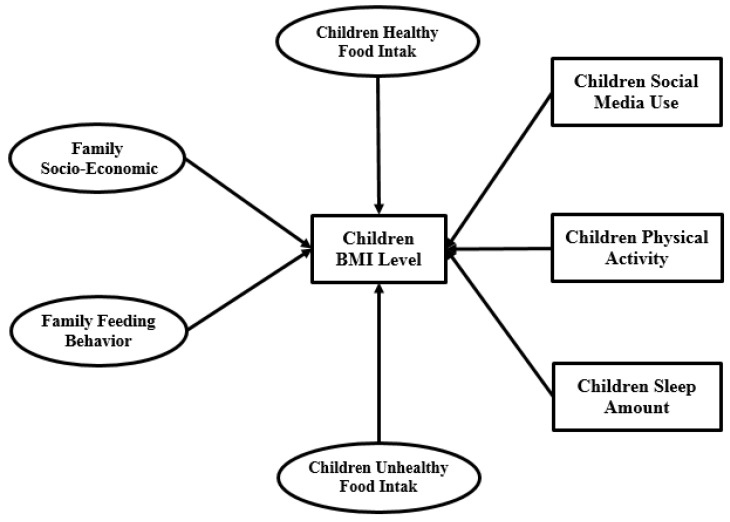
SEM research model.

Figure 5 illustrates the SEM research framework for both primary and high school students. Seven factors are considered as research model inputs. Four factors including children’s healthy food intake, children’s unhealthy food intake, family socio-economic status and family child-feeding behavior are latent constructs. The remaining factors, including children’s social media use, children’s physical activity and children’s sleep amount are measurement structures.

#### 4.2.1. SEM Analysis Validity and Reliability

Fornell, Larcker [58] defined the following terms and conditions for the reliability and validity of a questionnaire:

(a) Validity: for every latent variable the Cronbach’s alpha value must be equal to or higher than 0.7.
(b) Reliability:
  - The factor loading of every indicator of all latent variables must be higher than 0.70.
  - The average variance extracted (AVE) for every latent variable must be equal to or higher than 0.50.

**Figure 6:**
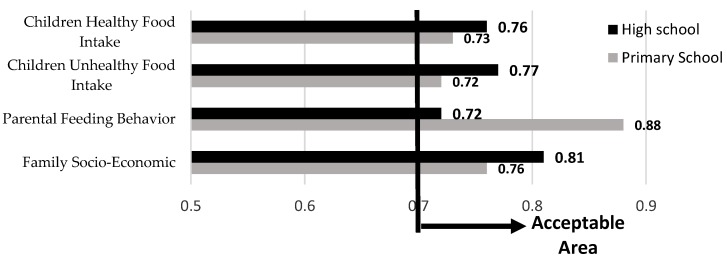
Cronbach’s alpha outputs.

Figure 6 represents the Cronbach’s alpha outputs of four latent variables for both primary and high school. Evidently, all indices are higher than 0.7. Therefore, the research model validity is accepted. 

Table 4 presents the factor loadings of children’s healthy food intake, children’s unhealthy food intake, family socio-economic status and family child-feeding behavior in both primary and high school models.

**Figure 7:**
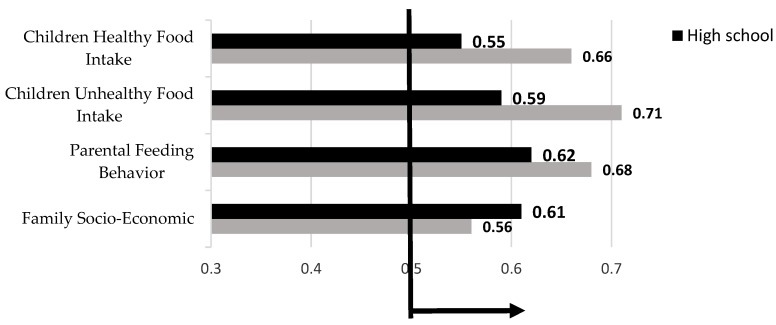
AVE analysis outputs.

Table 4 presents the factor loadings of the indicators of four research latent variables for both primary and high school separately. In the primary school model the age (father), age (mother), education (father), and modeling, and in the high school model the age (father), age (mother), rewarding, modeling and restricting have lower factor loadings (below 0.7). Therefore, these indicators must be excluded from the SEM analysis. By excluding some indicators, the study reliability is thus confirmed. Figure 7 presents the Average Variance Extracted (AVE) analysis outputs for both primary and high school levels. This figure illustrates that all research group variables have acceptable AVE values.

#### 4.2.2. Normality Testing

In SEM analysis, kurtosis and skewness are the most familiar indices for normality testing. If the absolute value of the kurtosis index is below 7 and the skewness index value is below 2, then the normality of the indicator is acceptable. Table 5 provides the normality test analysis of each variable for both primary and high school models. The normality of all indicators (which was accepted according to Section 4.2.1.) is accepted individually according to the skewness and kurtosis outputs. Furthermore, based on the multivariate normality test output, the kurtosis values are 8.391 and 9.023 for primary and high school. These values are lower than 10, and therefore the multivariate normality is accepted [59].

#### 4.2.3. Model Fitting

**Figure 8:**
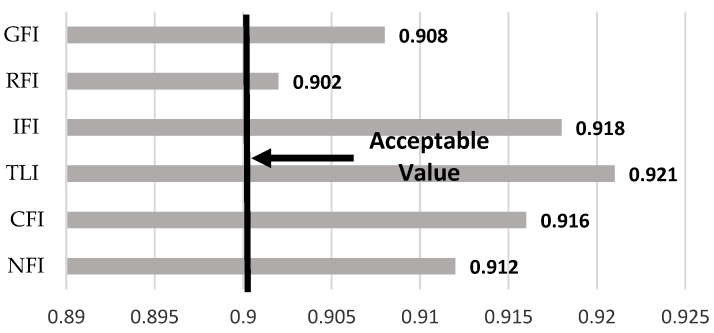
Model fit analysis.

Figure 8 shows the output of SEM model fitting. The goodness of fit index (GFI), relative fit index (RFI), incremental fit index (IFI), Tucker Lewis index (TLI), comparative fit index (CFI), and normed fit index (NFI) values are within acceptable ranges.

#### 4.2.4. Multicollinearity Analysis

**Figure 9:**
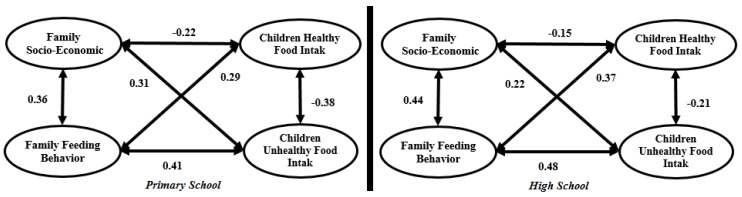
Full measurement model.

In SEM analysis, multicollinearity among latent variables is a serious problem. Weak discriminant validity of the research model employed usually causes multicollinearity. In Figure 9, the double arrow represents the covariance among latent variables. Kline and Klammer [60] determined that if the correlation between two latent variables is bigger than 0.85, the research model has a multicollinearity problem. Based on the SEM measurement model outputs presented in Figure 9, the correlation among the four latent variables does not exceed 0.85 for both primary and high school, which validates there is no multicollinearity in the current research model.

#### 4.2.5. Structural Model

**Figure 10:**
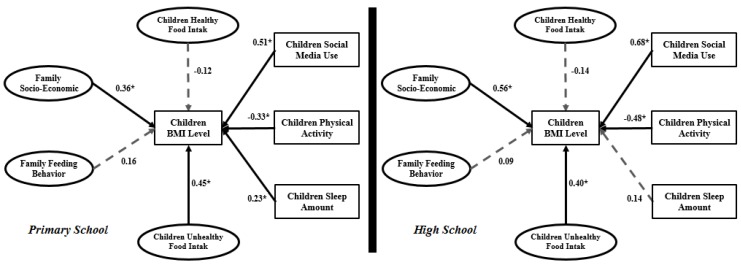
Structural primary and high school models.

A structural model is used to recognize the hypothesized relationship among research variables, which is linked to the presumed model’s conception. Figure 10 and Table 6 present the structural primary and high school models.

From the seven relationships in the primary school model, the impact of both family child-feeding behavior and children’s healthy food intake on child BMI is not significant. However, in the high school model, family child-feeding behavior and children’s sleep amount do not have a significant impact on child BMI. In the primary school model, children’s social media use (β = 0.51), family socio-economic status (β = 0.36) and children’s physical activity (β = −0.33) have the highest values. However, in the high school model, family socio-economic status and children’s social media use can be said to have the same severe impact on child BMI. 

### 4.3. Taguchi Method Analysis

In this part of the study the significant research variables were selected from the SEM analysis. Then the Taguchi experiment was designed, data was extracted from the main dataset and data analysis was done based on the Taguchi method. In the primary school model, two variables including family child-feeding behavior and children’s healthy food intake were eliminated from the Taguchi experimental analysis. Therefore, the Taguchi analysis for primary school entailed five variables: family socio-economic status, children’s unhealthy food intake, children’s social media use, children’s physical activity and children’s sleep amount. Every variable had five levels; therefore, the L_25_ (5^5^) Taguchi experimental design was applied. Thus, at least 25 participants must be extracted from the entire primary school dataset. In the high school model (Figure 10), three variables were excluded from the Taguchi experimental design, namely family child-feeding behavior, children’s healthy food intake and children’s sleep amount. Therefore, the Taguchi experimental design for high school involved four variables: family socio-economic status, children’s unhealthy food intake, children’s social media use, and children’s physical activity. In this part of the study, the L_20_ (4^5^) Taguchi experimental design with at least 20 participants from the high school dataset was used. MINITAB software was employed in this data analysis step. Table 7 contains the coding structure for data analysis with MINITAB software.

**Figure 11:**
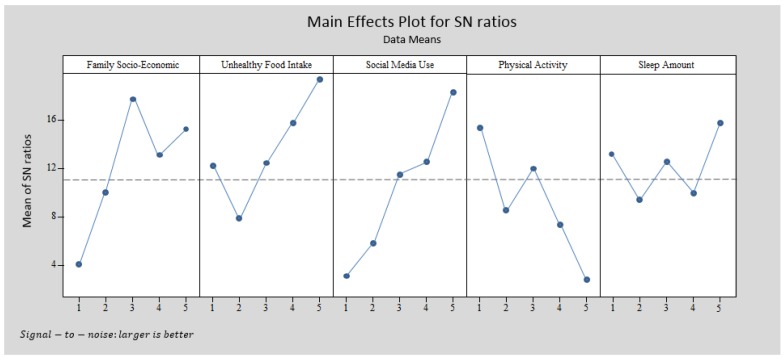
Taguchi output for the primary school obesity model with MINITAB software.

**Figure 12:**
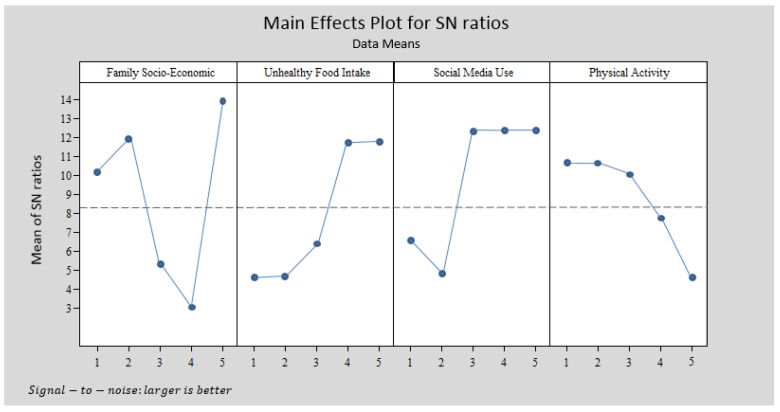
Taguchi output for the high school obesity model with MINITAB software.

Figure 11 and Figure 12 express the Taguchi method outputs from MINITAB software for primary schools and high schools.

The structures in Figure 11 and Figure 12 differ. The primary school pattern involved five variables and the high school pattern had four variables, which were chosen based on the SEM outputs. Figure 12 illustrates that the highest BMI occurred for families with moderate socio-economic status and children who always consume unhealthy food, use social media more than 4 h per day, have no physical activity and sleep more than 9 h per day. However, according to Figure 12, the highest BMI in the high school pattern was observed for children who mostly or always eat unhealthy food, use social media 2–3 h per day or more, have no physical activity or only once per week, and come from a family with very high socio-economic status.

## 5. Discussion

The main objective of this study was described in two main sections. The first section introduced an obesity model for primary and high school students by applying SEM. In this part of the study, the main effective indicators on the BMI of both groups were identified. The second section introduced a pattern based on the Taguchi method from the data and variables extracted from the data analysis in the first section and the Taguchi design process.

Based on the above objective, data was collected from 1569 participants (958 from primary schools and 611 from high schools). The research model introduced in Figure 5 includes four latent variables (family socio-economic status, family child-feeding behavior, children’s healthy food intake and children’s unhealthy food intake) and three measurement variables (children’s social media use, children’s physical activity and children’s sleep amount). This discussion contains two parts in terms of SEM and Taguchi method outputs.

Based on Table 2, primary school children have a 5.74% risk of becoming overweight. However, in the high school group the risk is 8.11%, which is higher than primary school (range: 8.11%–5.74% = 2.37%). Generally, 15.44% of primary school participants are obese and 19.14% of high school participants are overweight.

### 5.1. Discussion on SEM Outputs

According to the SEM analysis of the primary school model, *R*^2^ is 0.83. This means that 83% of child BMI variation is dependent on family socio-economic status, family child-feeding behavior, children’s unhealthy food intake, children’s social media use, children’s physical activity and children’s sleep amount. In the high school model the variation is 75% with significant effects from family socio-economic status, children’s unhealthy food intake, children’s social media use, and children’s physical activity. The *R*^2^ value is lower in the high school model than primary school. As mentioned before, the *R*^2^ value for the high school model is 0.75, which means that a 25% variation in BMI is related to other indicators that are not involved in the present research model.

#### 5.1.1. Obesity and Family Socio-Economic Status

Studies by Crouch et al. [61] and Walsh and Cullinan [62] confirmed that family socio-economic status has a significant impact on children’s weight. In the current study, child BMI was considered instead of weight. In both primary school and high school models, family socio-economic status has a significant impact on child BMI. However, the structure of significant indicators of family socio-economic status for primary school differs from high school. In both models, the parents’ age has no impact on family socio-economic status. Moreover, in the primary school model, the mother’s education has a lower factor loading. Therefore, these indicators were deleted from further data analysis (see Table 4).

#### 5.1.2. Obesity and Physical Activity and Sleep

Physical activity and amount of sleep have been considered in previous studies related to child obesity modeling [63]. These two indicators were included in the present research models. Physical activity has a negative significant impact on child BMI in both primary school (β = −0.33) and high school (β = −0.48) models. However, this impact is higher in the high school model. Children’s sleep amount has a significant impact on child BMI in the primary school model (β = 0.23) but not a significant impact in the high school model (β = 0.14). Therefore, the impact of children’s sleep amount and children’s physical activity in the primary school model differs from the high school model.

Two meta-analyses and systematic reviews related short sleep duration with obesity in children [64,65]. In the current study, it was found that the average daily sleep amount of children (primary school students) is significantly and positively related to BMI. For high school students, the impact of sleep amount is not significant on their BMI level. However, a study by Taheri, Lin [66] revealed that short sleep duration is related to alterations in metabolic hormones that promote energy intake. Another study by Spiegel, Tasali [67] confirmed that metabolic hormone alterations as a consequence of reduced amount of sleep is also associated with an increase in subjective hunger and appetite for unhealthy foods.

#### 5.1.3. Obesity and Social Media Use

In both primary school and high school models, children’s social media use has significant impact on child BMI. The impact in the high school model (β = 0.68) is much higher than in the primary school model (β = 0.51). Therefore, according to the high school model, students who spend more time using social media on devices like TV, video games, laptop/PC and mobile telephones exhibit a greater increase in BMI than primary school students. This variable with different definitions has been used in various studies. In this study, it was applied along with other important variables like healthy and unhealthy food intake, sleep amount, and physical activity. The SEM outputs confirm that in the primary school obesity model, children’s social media use has the highest impact (highest regression coefficient β = 0.51) among other input variables. Even two significant latent variables (i.e., family socio-economic status and children’s unhealthy food intake), which are a combination of some other indicators, have lower impact than children’s social media use (as one indicator). This indicator in the high school model is in second place after family socio-economic status with very little difference. Essentially, in both primary and high school obesity models, children’s social media use has the highest impact on child BMI. Therefore, it can be said that children’s social media use is a curtailing indicator of BMI increase in children.

As a result, the outputs from this study illustrate that increased daily use of social media is independently associated with greater BMI levels for both primary and high school students. This relation has been confirmed in a number of current studies [68,69,70,71]. However, according to the present study, the relationship between social media use and BMI level has the highest value among others. There are a few reasons for this result. Griffiths and Page [72] mentioned that obesity has been related to victimization and social isolation. Using technology may promote sedentariness and replace otherwise active behaviors, and may thus contribute to energy imbalance. Technology use has also been related to increased energy intake despite the absence of hunger, resulting in surplus energy intake in adolescents [73]. Moreover, Andreyeva et al. [74] approved that the advertising of unhealthy food like fast food and sugar sweetened beverages via technologies also affects the increased consumption of these foods among different generations, especially children and youth. These kinds of advertisements are not limited to TV, but are also found on the Internet and particularly in current mobile telephone applications. In current years, a number of energy drink companies have become main sponsors of some video games even. Adolescents and children are significantly immersed in these applications and become targets of optimum unhealthy beverage/food advertising. Our data analysis supports the notion that social media used with all kinds of technologies can significantly impact the increase in child BMI for primary and high school students.

#### 5.1.4. Comparison of SEM with Other Statistical Modeling

Three main advantages of SEM along with statistical disadvantages are presented below:

*Application of latent variables.* A particular advantage of SEM is the use of ‘latent variables.’ According to Bollen [75], “latent variables provide a degree of abstraction that permits us to describe relations among a class of events or variables that share something in common.” Latent variables refer to constructs that are not directly observable. However, the other technical modeling including regression and even the adaptive neuro fuzzy inference system (ANFIS) do not have this ability.
*The ability to perform simultaneous estimation.* There is a common limitation among correlation analysis*, t*-test, ANOVA, MANOVA and regression analysis. All these approaches can express a single correlation between the independent and dependent variables. In regression analysis, however, one or more independent variables are involved in a research model, but there should be only one dependent variable. In SEM it is possible to determine two or more dependent variables with different measurement and latent variable structures. MANOVA and canonical correlation may involve more than one independent and dependent variables, but analysis is restricted in that it is only possible to determine the linkage between independent and dependent variables. On the other hand, SEM can have the relationships among several dependent variables in a single model.
*SEM is able to estimate the direct and indirect effects in a single model.* Gefen et al. [76] believe that the most significant advantage of SEM technique is the ability to simultaneously model and examine the indirect and direct associations that exist among multiple independent and dependent variables.

### 5.2. Discussion of the Outputs from the Second Section

The outputs from the first section were used in the second section. Based on the SEM analysis outputs, the significant variables in obesity modeling were extracted and the Taguchi experiment was designed. The primary school model involved family socio-economic status, children’s unhealthy food intake, children’s social media use, children’s physical activity and children’s sleep. For the high school model, the family socio-economic status, children’s unhealthy food intake, children’s social media use, and child sleep amount were extracted (Figure 10). L_25_ (5^5^) and L_20_ (4^5^) were designed for the primary school and high school patterns. Figure 11 illustrates the MINITAB software output according to the Taguchi method design for primary schools. This figure represents a pattern which indicates that families with moderate socio-economic status and with children who always have unhealthy food intake, use social media more than 4 h per day, have no physical activity and sleep more than 9 h per day have children with the highest BMI. This pattern differs for high school students. The highest BMI in the high school pattern (Figure 12) is experienced by families with very high socio-economic status and with children who mostly or always eat unhealthy food, use social media 2–3 h per day or more, and have no physical activity or only once per day.

#### The Difference between the Taguchi Method and Other Statistical Modeling

The concept of data analysis with the Taguchi method is quite different from other statistical modeling, like regression, SEM, PLS and ANFIS. The output of modeling techniques is about the significance or non-significance of the independent variables in the research model. However, those outputs are not able to answer the following question:

The combination of what levels or categories of independent variables leads to dependent variable (higher or lower values) optimization?

Data analysis with the Taguchi method can answer the above question. The interpretation of Figure 12 is:

- The highest BMI in the high school pattern is observed for children who mostly or always eat unhealthy food, use social media 2–3 h per day or more, do no physical activity or only once per week, and come from a family with very high socio-economic status.
- The impact of two levels of unhealthy food intake (mostly and always) on BMI is the same.
- The impact of three levels of social media use (2–3 h per day, 3–4 h per day and more than 4 h per day) on BMI is the same.
- The impact of two levels of physical activity (none and 1–2 times per week) on BMI is the same.

According to the above discussion, it appears the Taguchi method is able to introduce a pattern of with a combination of different research variable levels. This feature is not feasible with statistical modeling, like regression, factor analysis, or even SEM, PLS and ANFIS.

## 6. Conclusions

There are currently serious overweight and obesity problems worldwide. Hence, researchers in this area seek to understand the effective variables and introduce solutions to control the BMI. Previous studies have utilized a range of methodologies to analyze overweight, such as descriptive statistics including diagrams, tables and charts, other modeling methods like regression and SEM, or even nonparametric analysis using fuzzy sets. This study combined SEM with the Taguchi method to introduce an improved pattern for public health studies in order to better understand the obesity levels of primary and high school students. The SEM output yielded significant effective variables on child obesity and the Taguchi method introduced a pattern based on a combination of significant variables that lead to high BMI in children.

## Figures and Tables

**Table 1:** BMI categories for children.

BMI Value	Category
<18.5	Underweight
18.5–22.9	Normal Range
23.0–24.9	At Risk
25.0–29.9	Moderately Obese
≥30.0	Severely Obese

**Table 2:** Body mass index (BMI) distribution.

Category	Primary School Number (%)	High School Number (%)
Underweight	115 (12%)	92 (15.1%)
Normal Range	695 (72.6%)	402 (65.8%)
At Risk (Overweight)	55 (5.7%)	50 (8.1%)
Moderately Obese (Overweight)	51 (5.3%)	38 (6.2%)
Severely Obese (Overweight)	42 (4.4%)	29 (4.8%)

**Table 3:** Distributions of parents’ characteristics (primary school and high school).

Father	Primary School (Number; %)	High School (Number; %)	Mother	Primary School (Number; %)	High School (Number; %)
Age of Parents					
Below 31 years old	26 (2.7%)	2 (0.3%)	Below 31 years old	59 (6.2%)	4 (0.7%)
31–40 years old	248 (25.9%)	55 (9%)	31–40 years old	255 (26.6%)	225 (36.8%)
41–50 years old	502 (52.4%)	304 (49.8%)	41–50 years old	363 (37.9%)	242 (39.6%)
51–60 years old	122 (12.7%)	221 (36.2%)	51–60 years old	248 (25.9%)	132 (21.6%)
Over 60 years old	60 (6.3%)	29 (4.7%)	Over 60 years old	33 (3.4%)	8 (1.3%)
Job Experience of Parents					
Less than 5 years	25 (2.6%)	7 (1.2%)	Less than 5 years	9 (0.9%)	12 (2%)
5–10 years	126 (13.1%)	136 (22.2%)	5–10 years	269 (28.1%)	263 (43%)
11–15 years	498 (52%)	402 (46.7%)	11–15 years	402 (42%)	189 (30.9%)
16–20 years	222 (23.2%)	252 (24.7%)	16–20 years	252 (26.3%)	116 (19%)
More than 20 years	87 (9.1%)	26 (5.2%)	More than 20 years	26 (2.7%)	31 (5.1%)
Income of Parents					
Less than 2MT per month	20 (2.1%)	7 (1.1%)	Less than 2MT per month	102 (10.6%)	75 (12.3%)
2MT–3MT per month	76 (7.9%)	48 (7.9%)	2MT–3MT per month	558 (58.2%)	235 (38.5%)
3MT–4MT per month	333 (34.8%)	268 (43.9%)	3MT–4MT per month	151 (15.8%)	109 (17.8%)
4MT–5MT per month	285 (29.7%)	252 (41.2%)	4MT–5MT per month	108 (11.3%)	170 (27.8%)
More than 5MT per month	244 (25.5%)	36 (5.9%)	More than 5MT per month	39 (4.1%)	22 (3.6%)
Education Level of Parents					
Less than high school	44 (4.6%)	11 (1.8%)	Less than high school	25 (2.6%)	18 (2.9%)
High School	48 (5.0%)	22 (3.6%)	High School	152 (15.9%)	85 (13.9%)
Diploma	550 (57.4%)	336 (55%)	Diploma	335 (35%)	295 (48.3%)
Bachelor	258 (26.9%)	222 (36.3%)	Bachelor	351 (36.6%)	201 (32.9%)
Master or PhD	58 (6.1%)	20 (3.3%)	Master or PhD	95 (9.9%)	12 (2%)

**Table 4:** Factor loading analysis of the research latent variables.

Parameter Description	Factor Loading Primary School	Factor Loading High School
Family Socio-Economic		
Age (Father)	0.56	0.49
Age (Mother)	0.61	0.59
Education (Father)	0.52	0.71
Education (Mother)	0.81	0.77
Income (Father)	0.92	0.88
Income (Mother)	0.73	0.72
Job Experience (Father)	0.82	0.83
Job Experience (Mother)	0.74	0.76
Parental Feeding Behavior		
Rewarding	0.71	0.65
Restricting	0.78	0.52
Pressuring	0.79	0.76
Modeling	0.66	0.49
Controlling	0.81	0.88
Monitoring	0.76	0.79
Children Unhealthy Food Intake		
Sweets	0.78	0.82
Chips	0.79	0.86
Soft Drinks	0.74	0.76
Fast Food	0.82	0.79
Children healthy Food Intake		
Vegetables	0.75	0.83
Fruits	0.81	0.72
Whole Grains	0.88	0.73

**Table 5:** Normality test.

Indicators	Primary School		High School	
	Kurtosis	Skew	Kurtosis	Skew
Age (Father)	Deleted from the model		Deleted from the model	
Age (Mother)	Deleted from the model		Deleted from the model	
Education (Father)	Deleted from the model		3.65	1.76
Education (Mother)	1.25	0.98	2.69	0.58
Income (Father)	–2.36	–0.55	1.54	0.98
Income (Mother)	1.52	1.06	2.11	1.19
Job Experience (Father)	5.25	1.66	2.58	1.03
Job Experience (Mother)	3.61	1.19	–0.95	–0.11
Rewarding	–3.84	–1.18	Deleted from the model	
Restricting	0.44	0.85	Deleted from the model	
Pressuring	2.41	1.09	1.09	0.26
Modeling	4.59	1.55	Deleted from the model	
Controlling	–3.81	–1.13	2.44	1.01
Monitoring	2.99	1.49	1.36	0.55
Sweets	1.91	1.02	1.67	1.47
Chips	3.17	1.27	–0.28	–1.03
Soft Drinks	2.77	1.92	–0.18	–0.08
Fast Food	3.55	1.83	2.33	0.99
Vegetables	2.93	1.73	–3.33	–0.95
Fruits	4.76	1.44	2.01	1.88
Whole Grains	–3.93	–1.79	3.57	1.82
Children’s Social Media Use	2.68	1.76	–3.22	–1.31
Children’s Physical Activity	2.56	1.24	3.09	0.27
Children’s Sleep Amount	3.29	1.66	–1.94	–1.08

**Table 6:** SEM analysis outputs for primary and high school students.

Independent Variables	Beta	z-Value	*p*-Value	95% CI
Primary School				
Family Socio-Economic Status	0.36	3.90	<0.01	(0.29, 0.42)
Family Feeding Behavior	0.16	1.73	0.08	(0.09, 0.21)
Children’s Healthy Food Intake	–0.12	1.30	0.12	(–0.18, 0.01)
Children’s Unhealthy Food Intake	0.45	4.87	<0.01	(0.33, 0.61)
Children’s Social Media Use	0.51	5.52	<0.01	(0.44, 0.58)
Children’s Physical Activity	–0.33	3.57	<0.01	(–0.41, –0.22)
Children’s Sleep Amount	0.23	2.49	0.02	(0.14, 0.31)
High School				
Family Socio-Economic Status	0.56	6.06	<0.01	(0.43, 0.70)
Family Feeding Behavior	0.09	0.97	0.56	(–0.07, 0.18)
Children’s Healthy Food Intake	–0.14	1.52	0.11	(–0.21, –0.02)
Children’s Unhealthy Food Intake	0.40	4.33	<0.01	(0.31, 0.46)
Children’s Social Media Use	0.68	7.36	<0.01	(0.59, 0.77)
Children’s Physical Activity	–0.48	5.20	<0.01	(–0.52, –0.39)
Children’s Sleep Amount	0.14	1.52	0.13	(0.06, 0.19)

**Table 7:** Taguchi method coding.

Level	Coding	Level	Coding
Family Socio-Economic Status		Children’s Social Media Use	
Very Low	Code “1”	Less than 1 h per day	Code “1”
Low	Code “2”	1–2 h per day	Code “2”
Moderate	Code “3”	2–3 h per day	Code “3”
High	Code “4”	3–4 h per day	Code “4”
Very High	Code “5”	More than 4 h per day	Code “5”
Children’s Unhealthy Food Intake		Children’s Physical Activity	
Never	Code “1”	None	Code “1”
Rarely	Code “2”	1–2 times per week	Code “2”
Sometimes	Code “3”	3–4 times per week	Code “3”
Mostly	Code “4”	5–6 times per week	Code “4”
Always	Code “5”	Every day	Code “5”
Children’s Sleep Amount			
Less than 6 h per day	Code “1”		
6–7 h per day	Code “2”		
7–8 h per day	Code “3”		
8–9 h per day	Code “4”		
More than 9 h per day	Code “5”		
